# Rethinking GLAMs as commons: a conceptual framework

**DOI:** 10.12688/openreseurope.16473.2

**Published:** 2024-07-09

**Authors:** Vasilis Avdikos, Mina Dragouni, Martha Michailidou, Dimitris Pettas

**Affiliations:** 1 Department of Economic and Regional Development, Panteion University of Social and Political Sciences, Athens, Attica, 17671, Greece; 2 Department of Communication, Media and Culture, Panteion University of Social and Political Sciences, Athens, Attica, 17671, Greece

**Keywords:** commons, GLAMs, heritage, museums, governance, cultural policy

## Abstract

In this paper, we reflect on ‘new commons’ theory and elaborate on the ontology of memory institutions with the aim to develop a comprehensive conceptual framework for ‘commons-oriented’ GLAMs (Galleries, Libraries, Archives and Museums). In doing so, we propose an alternative for governing and managing cultural heritage against a backdrop of limited public funding and pressures for developing memory institutions into more inclusive, participatory and ‘entrepreneurial’ organisations. Our theoretical analysis is grounded in the review of the extant literature, including both the ‘Ostromian’ and ‘Autonomist’ schools of thought, drawing particularly on the new commons (urban, digital, cultural/heritage commons) to inform our framework. The conceptual schema we present here is adapted to fit with the idiosyncrasies of the sector, describing the functioning of commoning practices in GLAMs. Such a framework is critical for navigating future research and devising workable solutions to address current challenges for memory institutions in Europe.

## Plain language summary

Over the last decades, GLAMs (Galleries, Libraries, Archives and Museums) have faced several challenges, including the limitation of public funding due to the global economic crisis of 2008, the need to keep up with digitalization trends in order to make collections accessible to larger audiences, the implications of the COVID-19 pandemic which impacted income sources such as visitor revenues, licensing, donations, endowments and sponsorships. All these challenges require GLAMs to be proactive in adopting innovative strategies, collaborating with other institutions, leveraging partnerships, and seeking sustainable funding models to address their unique and evolving needs. In this paper, we explore the potentiality of GLAMs to operate as commons, towards ensuring their vibrancy, sustainability and resilience, while meeting broader societal needs. The commons constitute a mode of resource management (in our case cultural ones) through which a specific community (instead of a state body or actors of the market) is responsible for the exploitation and managmenet (or, in several cases also the production) of a resource under inclusive and democratic principles. In this way, the commons constitute of i) a (set of) commons pool resource(s), including a diverse range of material and intangible resources, ii) a group/ community that appropriate, use, manage and take care of the resources, iii) a governance/ management framework established through mixes of official and informal sets of rules, decision-making processes, governance arrangements.

## Introduction

The outbreak of the COVID-19 pandemic found a vast number of European memory institutions pressured by understaffing and limited funding ( ICOM, 2020; Montalto *et al.,* 2020). According to Pessach (2008) ‘memory institutions are social entities that select, document, contextualize, preserve, index, and thus canonize elements of humanity’s culture, historical narratives, individual, and collective memories’. Such organisations that make up much of the ‘GLAM’ sector (Galleries, Libraries, Archives and Museums) have traditionally served as ‘reservoirs’ of knowledge and custodians of heritage. Yet, as prescribed by international conventions and current European policy (see *inter alia* Council of Europe, 2022; European Commission, 2014; European Union, 2019; UNESCO, 2015), today, GLAMs are expected to assume a central role in society, promote participation and inclusion, follow digitisation trends and ‘justify’ their public character through wide access and representation across diverse audiences.

The COVID-19 pandemic served as a wake-up call to rethink how memory institutions produce their work and bring it to the public, reflecting on potential ways to develop their resilience in precarious times. The key question is whether and how, memory institutions could ensure their sustainable operation by increasing their financial autonomy and securing resources without sacrificing their societal mission. Ideally, achieving a greater integration with surrounding communities and strengthening their profile as ‘open’ institutions. Considering on the one hand, the cutting down of public funding for the culture and heritage sector due to the austerity policies implemented in the aftermath of the economic crisis of 2008 ( Romolini *et al.,* 2020), and on the other hand, the inherent limits of the (profit-seeking) neoliberal business models to serve collective benefit, it becomes obvious that we need to consider alternatives that move away from the state-market dilemma and dare to think of an alternative paradigm; that of the commons.

Towards this direction, in this paper, we review the extant literature of the commons in order to elaborate on the ontology of GLAMs and heritage commons and develop a conceptual framework of ‘commons-oriented’ GLAMs to navigate future research for workable solutions to the sector. Our review features the ‘Ostromian’ understandings of the commons, insights from the autonomist school of thought and other theoretical and practical articulations of the ‘new commons’. By exploring different types of commons-based ventures, such as urban commons, digital commons and cultural/heritage commons, we distinguish those elements that are mostly relevant to bring on board. Based on these we develop and present here, for the first time, a novel conceptual framework for studying GLAMs as commons, adapted to fit with the idiosyncrasies of the sector. Moreover, we explore the ways relevant commoning practices could be developed towards ensuring GLAMs’ sustainability and resilience, while meeting broader societal needs. According to Linebaugh (2008), the social process, the praxis of commoning, refers to the collective management of resources. Furthermore, the creation and reproduction of the commons is materialised through commoning practices of co-production, co-appropriation and co-management, developed upon horizontal and democratic principles and processes ( Card, 2018).

## The new commons: a literature review

In recent years, the concept of the commons has emerged in academic, public and policy debates, as a solution for managing natural, urban and intangible resources in a collective, democratic, inclusive and sustainable way and responding to contemporary social, economic and environmental challenges. Since the 1990s, commons have attracted much scholarly attention, led by the work of Elinor Ostrom ( Ostrom, 1990; Ostrom & Hess, 2007). Although these early debates revolved around natural resources and their collective management, the discussion soon opened-up to a variety of resources of different attributes and qualities, from public spaces and urban infrastructure to knowledge and digital archives (e.g. Benkler, 2017; Dolšak & Ostrom 2003; Foster & Iaione, 2016; Lessig, 2003; Parker & Johansson, 2011).

The Ostromian approach was very influential in commons scholarship and made a major contribution to related theory by i) conceptualising the commons as social systems, where resources are just one component, ii) positioning the commons as an alternative to public/ private ownership and control and iii) providing evidence that the viability, sustainability and efficient management of the commons do not presuppose specific property regimes: community-owned, public, private, semi-private or mixed regimes can all support the longevity of common social systems ( Arvanitidis, 2017; Arvidsson, 2019; Gibson-Graham *et al*., 2013; Williams, 2018).

Although Ostrom’s contribution is widely acknowledged and undisputed, it has been argued that the Ostromian approach is limited by overlooking the role of political antagonism, power relations and political conflicts ( Kioupkiolis, 2020b). The ‘autonomist school’ of commons that emerged in the early 2000s, shifted the focus from empirical studies concerned merely with management to issues of power and the prefigurative and transformative potentials of the commons (see *inter alia* De Angelis, 2007; De Angelis, 2017; Hardt & Negri, 2000; Hardt & Negri, 2004; Hardt & Negri, 2009; Holloway, 2010; Stavridis, 2016). This second stream of commons thought ( Huron, 2015) was imbued with a vision of emancipation realised through the emergence of new collective subjectivities ( Hardt & Negri, 2009) that could produce alternative 'institutions' ( Federici & Caffentzis, 2013; Harvey, 2012), whose strategic alliances would promote commons as a 'shared project' that challenges dominant power relations and creates space for autonomous practice and transformation ( Hardt & Negri, 2012; Hardt & Negri, 2017). In parallel, research on the commons also expanded its focus to include social systems that emerge around resources beyond the ‘traditional’ ( Parker & Johansson, 2011), such as urban, digital and cultural commons. Compared to natural resources, these ‘new commons’ are mostly non-subtractive and non-excludable ( Benkler, 2017), whereas their usage and consumption are intertwined with their production and reproduction. In the paragraphs that follow we present each of these types of new commons along with their key characteristics.

### The urban commons

The urban commons are spaces of public interest that are catered and managed by communities. They are developed around a diverse range of material and intangible resources, including public infrastructure (e.g., gas and electric distribution systems) ( Brain, 2019; Garnett, 2012; Łapniewska, 2017; Lee & Webster, 2006; Löfgren, 2015; Newman, 2013), housing, modes of production, consumption and social reproduction, including social services, community gardens, food networks, transportation ( Borgström *et al*., 2006; Bruun, 2015; Federici, 2010; Parr, 2015; Sardeshpande *et al*., 2021; Susser & Tonnelat, 2013) and urban waste ( Zapata & Zapata, 2015).

For Huron (2015), the urban commons are characterised by two unique attributes. Firstly, they emerge and operate in a saturated space, namely spatial units which are “already densely packed with people, competing uses, and capitalist investment”. Secondly, they are created and enabled by groups of strangers that come together. For Bauwens & Niaros (2017), the urban commons create new sets of challenges within the urban environment. These include among others i) a claim on behalf of citizens to manage resources outside the public-private dichotomy and a shift from representative to contributory democracy and, ii) the challenging of market power, through the emergence of a generative economy that is not extractive towards nature and humans.

Foster & Iaione (2016), argue that the commons is a claim to a resource. Such claims bring out the broader social value or utility that a specific resource can generate for urban communities through the granting of rights to the collective access and use and the overcoming of exclusionary conditions that could be imposed under private or public control. This association has been further developed by Eidelman & Safransky (2020), seeing urban space as urban commons through its collective management and autogestion that prioritises use values over exchange values. For Eidelman & Safransky (2020) urban commons is a political claim whereby ‘the right to the city encompasses the right to access, inhabit, and use urban space as well as the right to shape and govern it in transformational (anti-capitalist) ways’. In the same vein, Susser & Tonnelat (2013) associate the urban commons with different manifestations of the right to the city: i) the right to urban everyday life, developed around issues of production, consumption, access to public goods and services, ii) the right to simultaneity and encounters, developed around public spaces and the public sphere (streets, transportation and digital infrastructure etc.) and iii) the right to creative activity, revolving around collective visions of the cities through the activity of creative collectives and groups (see also Harvey, 2012; Narotzky, 2013). Cultural and memory institutions (GLAMs) can play a central role in reorganising the urban layout of cities, providing public access to their heritage resources that can serve as ‘raw materials’ for fostering creativity.

### The digital commons

In recent years, new information and communication technologies have served as drivers for the emergence and crystallisation of the ‘new commons’ paradigm in the digital realm. The ‘digital commons’ feature open knowledge, software and design (virtual) resources that ‘are the fruit of the labour of communities which reside in cyberspace’ ( Dafermos, 2020). Digital commons harness peer-to-peer (P2P) practices to create and maintain open and shared resources through a commune logic ( Bauwens *et al*., 2019). In doing so, digital commons popularise a new economic paradigm of co-operation that produces value through openness, sharing and global networks ( Tapscott & Williams, 2008).

Digital information, as well as access to digital tools and infrastructure, represents a non-excludable and non-rival public good. As such it can either be commodified and enclosed or produced as a commons and distributed under open licensing terms ( Dulong de Rosnay & Le Crosnier, 2012). The free and open source software are characteristic examples of goods circulating under an open access regime, allowing anyone to copy, use, modify and redistribute modified codes ( Benkler, 2016). This is facilitated greatly by the characteristics of the produced goods in question; digital commons are not simply non-competitive but even more so, they are goods where the more they are used, the more the benefits they yield for their users ( Kioupkiolis, 2022). Organised around virtual communities of creators and users (e.g. computer scientists, software developers, researchers, artists), digital commons feature community network and digital commons projects, free software, digital content (under non-commercial Creative Commons licences) and digital platforms ( Fuchs, 2021).

Equally interesting is the mode of decentralised production and co-operation that is pioneered in digital commons through the P2P model. Peer production represents a socio-technical system where large groups of individuals cooperate asynchronously as producers (of information, knowledge, culture) by ‘skipping’ market pricing and managerial hierarchies ( Benkler & Nissenbaum, 2006; Kostakis *et al*., 2018). Thus, peer production does not merely describe a new technological infrastructure or mode of production but a whole new set of interactions and social relations ( Papadimitropoulos, 2020). As Kioupkiolis (2022) observes, cyberspace hosts communities of commoners that are open and inclusionary but at the same time fragmented, heterogeneous and geographically unbound.

The digital commoning patterns of collaboration and collective self-government hold tremendous potential to foster the democratic values of plurality, participation, mutuality and openness ( Kioupkiolis, 2022: 52), all aligned to GLAMs’ current agenda. In fact, both official policy (e.g. Europeana’s Public Domain Charter) and civil society/grassroots initiatives, such as the Open GLAM movement, that emerged in the sector during the 2010s have sought to establish openness of digitised collections and artwork, as good professional practice for GLAMs digital work ( Sanderhoff, 2014). The importance assigned to openness and to defending creative work in the Public Domain make digital commons particularly relevant to the GLAM sector and to memory institutions, whose resources are undergoing a digital transition.

### The cultural commons and heritage commons

Cultural/heritage commons are often identified as a broad range of tangible and intangible cultural elements that are shared by a community, such as cultural traits, traditions and practices ( Bertacchini *et al*., 2012; Edwards, 2015; Eriksen, 2019; Santagata *et al*., 2011). The term is often used interchangeably with ‘knowledge commons’ and ‘information commons’ as pools of ideas, research, and innovation ( Hess, 2012; Madison *et al*. 2010). As suggested by Pelissier (2021: 3–4), cultural commons, such as public libraries, enable creative practices to sustain an ‘ecosystem that facilitates and democratises popular expression’.

In recent years, a strand of the literature has focused on the new possibilities opened-up by digital media to encourage collective action for the creation of new cultural resources as cultural commons. Some examples include the work of Carbone & Trimarchi (2012) on ‘Twitter Opera’ hosted by London’s Royal Opera House, the work of Marttila & Botero (2017) on community digital tools and Dalla Chiesa (2020) on crowdsourcing/crowdfunding as bottom-up cooperation protocols in the cultural sector. Although GLAMs are increasingly experimenting with community-curated exhibits and projects, they are still not necessarily community-driven, especially when audiences are invited to take part in an institutionally-controlled process ( Simon, 2010). These approaches have been criticised for using commons theory to describe projects that are participatory but not actually ‘commons’ (e.g. Iaione *et al*., 2022; Lekakis, 2020a). As Dalfovo (2020) stressed, coining participatory approaches as ‘cultural commons’ risks the danger of unwillingly encouraging ‘commons-washing’ practices by ‘feeding the misleading paradigm of creative and cultural processes being the output of voluntary actions carried out for one’s own pleasure and in one’s own free time’ without the respective compensation of mutual benefits.

At the same time, the commons framework has allowed researchers to analyse the susceptibility of cultural and heritage resources to enclosures (e.g. by market interests or political rhetoric), exclusion/inclusion patterns and the inefficiency of traditional policy and management patterns to fulfil the public function of related goods. For example, Gonzalez (2013) discussed how gentrification mechanisms and real-estate/tourism rents capture the (shared) value of heritage sites (as commons resources). Eriksen (2019) saw cultural commons as threatened by commercialisation and the misuse of cultural meaning by market forces. Bertacchini (2020) voiced his concerns with heritage ‘disneyfication’, stemming from conflicting uses and state patronisation that impose restrictions on access.

In this light, heritage commons have been envisioned as dynamic and porous systems that protect the resources at hand while catering for the common benefit of involved communities ( Lekakis, 2020a). In such systems, cultural heritage can be understood as a resource that is compiled collectively, as part of the work performed by memory institutions operating in the GLAM sector, to promote greater representation and more democratic/multivocal discourses about the past. In line with other commons, this ‘collective production’ of cultural/heritage commons can bring together a community of commoners ( Bailey & Marcucci, 2014). But, as scholarly work has emphasised, ‘communities’ (geographic, virtual or imaginative) are not always ‘community-like’ but rather incohesive assemblages of people with divergent interests ( Waterton & Smith, 2010). This, these communities with interest in the cultural/ heritage resource shall be regarded as plural and geographically-unbound. and they may include culture professionals, art workers, heritage researchers, local residents, patrons and many others ( Bailey & Marcucci, 2014). These communities are likely to be formed dynamically, reacting against the neglect or even hostility of public authorities (e.g. Teatro Valley in Italy; Bailey & Marcucci, 2014), or they may take a proactive approach to heritage-making (e.g. grassroots oral history projects, such as the ‘Oral History Groups’ in Greece ^1^).

More fundamentally, and beyond the relations of communities to other, ‘external’, actors, as Bruncevic ( 2018, p. 143) argues in her analysis of the fluid and contested nature of community and property of cultural artefacts “one of the more significant problems with the concept of community is that it is often presupposed as homogeneous or at least as an, at any given point, definable group”. As, however, heterogeneity of actors and potential divergence of interests are characteristic of contemporary complex communities, conflict within the community over what may be considered cultural resources and how these may be optimally managed becomes a central issue to be addressed through collective commoning practices. Recognising that divergence and adversarious positions are inherent in communities, we may conceive of “agonistic pluralism” ( Mouffe, 1999; Mouffe, 2007) as an essential element of community building, and recognise cultural commons as “agonistic commons” ( Kioupkiolis, 2019), which could “nurture bonds of mutual recognition and coalescence among different parties who remain rivals along certain dimensions, making more space for plural freedom under conditions of dissent and division” ( Kioupkiolis, 2019, p. 28). It is not, therefore, the existence of divergence and adversariality per se which is antithetical to the notion and practice of community, or to the practices of commoning devised by a community, but the management of such divergence in ways which will not presuppose the homogeneity or even uniformity of participating actors and entities. This brings to the fore the importance of institutions and processes of governance.

Moreover, Barrère (2018) suggests that cultural heritage resources operating as cultural commons ‘have to be managed through common institutions’. Following Ostrom’s theory, ‘institutions’ can be understood as encompassing formal and informal sets of rules, from laws and regulations to unofficial agreements or shared practices/traditions, that cannot be imposed from the outside but rather emerge organically from inside the common in line to the social, cultural and political traditions of the commoners ( Gould, 2017). For Gould (2017), a cultural commons conceptual framework needs to focus on those institutions that regulate activity (i.e. production, use and management) and therefore, a more in-depth enquiry into governance, ‘the central issue in commons scholarship’, would be critical for suggesting a way forward ( Gould, 2017).

Overall, despite the growing interest in conceptualising culture and heritage as commons, there is still relatively limited work in the organisation and management of related resources as commons by memory institutions, such as GLAMs ( Gould, 2017; Lekakis, 2020a; Lekakis & Dragouni, 2020). It is therefore necessary to delve deeper into the governance/management patterns and the design principles of cultural/heritage commons in order to address the ontology of these systems (i.e. resources, communities and rules of organising everyday work and strategy). This would be vital for informing our enquiry of how GLAMs could function as commons-oriented organisations. Yet, to avoid overestimating GLAMs’ potential to be governed as commons while underestimating managerial and political realities of their operation - from funding and long-term engagement to control and power issues over the uses of the past - it would be important to consider some of their distinctive features (e.g. organisation size, broader networks and relationships)..

## Situating the GLAMs in the commons literature

The aforementioned literature around the new commons largely informs the conceptual framework for commons-oriented GLAMs presented in this paper, as GLAMs i) comprise institutions, networks and initiatives that operate within the cultural sector, ii) growingly employ digital means and infrastructure in their collections management, content creation and outreach activities, iii) similarly to urban commons, they increasingly reach out to and engage with visitors/users and broader communities, such as civil society, citizens’ groups and, social movements in various aspects of their work, untapping their potential to serve as terrains where under-privileged groups can claim visibility and shed light upon neglected or oppressed sides of urban history and collective memory.

From an ontological standpoint, we may conceive GLAMs as a category of new commons, embodying characteristics primarily of the cultural/heritage commons but also of the urban and digital commons. A key distinction between ‘traditional’ and new commons lies in their subtractive/non-subtractive and excludable/non-excludable character. There are several cultural and heritage resources, such as cultural creations and their digital reproductions that share characteristics with knowledge/information commons, such as abundance and anti-rivalry. These cultural goods can be treated as infinite intellectual resources that (contrary to common-pool resources) may be challenged by underproduction/under-consumption and stagnation ( Bertacchini *et al*., 2012). Under-use in the realm of GLAMs may be expressed as limited numbers of visitors to physical exhibitions, archival or book collections, low attendance at art performances, workshops and cultural events, as well as sporadic engagement in digital platforms, repositories or applications. Thus, in GLAMs, resources are non-subtractive, while they are characterised by different degrees of excludability. The same holds for GLAMs’ digital or digitised resources, where consumption does not lead to depletion but rather to invigoration.

Although digitising collections can be an expensive and time-consuming process, materials can be reproduced at low or zero marginal cost. In several cases, intense use can lead to the attachment of added value to the resource, may it be societal (e.g. an openly accessible scientific paper in a library’s digital repository), or economic (e.g. an artefact in a gallery) (see also European Commission, 2015; Güner & Gülaçtı, 2022). As for excludability, the status is determined by several factors, including the character of the resource and, most importantly, the rules that define access to it. For example, an openly accessible digital resource (under CC0 licence) is de jure non-excludable, as well as artwork that has entered the Public Domain. On the other hand, an exhibition in the physical space of a museum, besides given restrictions concerning the spaces’ capacity, is primarily defined by the admissions’ regime and organisation policy of each GLAM (often directly related to ownership and legal status, aims and goals, and funding etc.). For example, membership schemes and copyright restrictions to digitised imagery can limit access and use by some social groups.

The three key components that are identified in commons literature (resources, communities, governance and management arrangements) are also present in the GLAM sector. In this paper, we explore the ways these components, brought together by sets of commoning practices, could be assembled towards establishing what we frame as commons-oriented GLAMs. We use the commons perspective as a prefigurative solution for the further thriving and sustainability of GLAMs, particularly the smaller and community-led memory organisations that operate in peripheral areas and have greater flexibility to adopt and adapt commons-like arrangements.

## A conceptual framework for commons-based GLAMs

**Figure 1.**
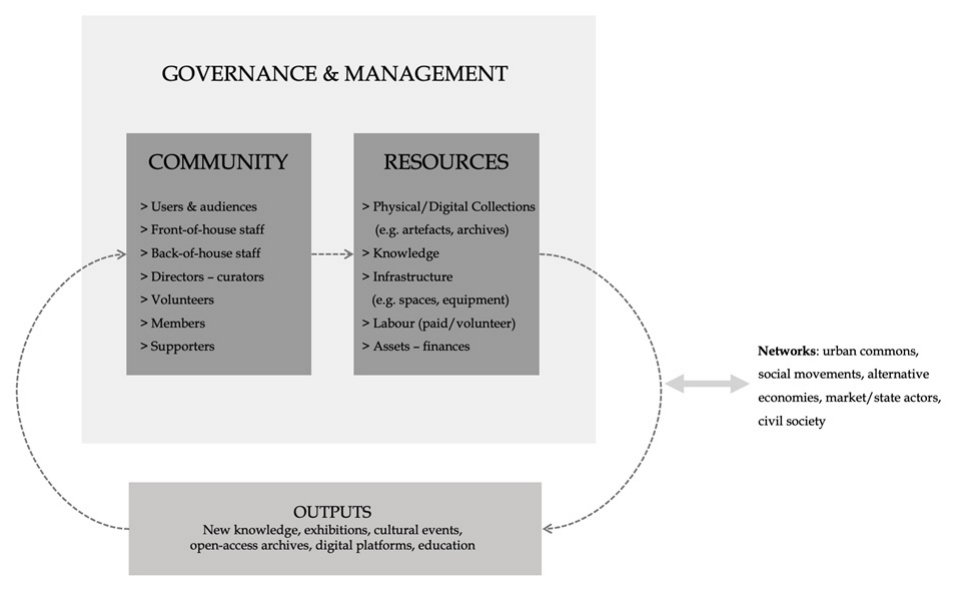
Common-oriented GLAMs as porous ‘circuits’.

Similarly to new commons, commons-based GLAMs can be analysed through the tripartite schema of a commons’ resource, a self-governing community and a set of self-legislated rules and norms that tune the commoning process (e.g. access/use, benefits distribution, financing). We further this tripartite schema, by emphasising its porous and circuit flows (see Figure 1). We explore GLAMs’ potential to operate as open terrains where various components (e.g. people, artefacts, information, spaces, capital) come together and assemble into a self-governed entity with a shared interest over the protection and dissemination of a common cultural/heritage resource. We further argue that in order to introduce, mobilise and continue reproducing a ‘commons’ logic, GLAMs need to untap their transformative capacity. As shown in Figure 1, we conceptualise commons-oriented GLAMs’ arrangements, daily operation and practices of inclusion as part of broader flows and networks, resting inside and outside of the sector, together constituting a commons’ ‘social system’.

Firstly, the ‘resources’ of a GLAM can be material or intangible, physical or digital, varying from collections and archives of artefacts, data, infrastructure (physical spaces, digital platforms etc.) to different modes of labour (waged, volunteer) and to sector-specific (e.g. conservation practices) and content-specific (e.g. particular heritages and pasts) collective knowledge. This aligns with the resources’ typology proposed by Ostrom & Hess (2007) for knowledge commons. Yet, we need to bear in mind that contrary to traditional commons, in commons-oriented GLAMs, the emphasis is placed on the community (and its memory, well-being etc.), not on the resource per se (i.e. a piece of art). This fits well with current theory and good practice in heritage management, advocating for a change of paradigm towards more human-centric practice that deviates from the protection of heritage as static material outside society (see for instance, the Burra Charter, ICOMOS, 2013; the Faro Convention, Council of Europe, 2005; the Intangible Cultural Heritage Convention, UNESCO, 2003).

Secondly, GLAMs’ communities of ‘commoners’ are plural and porous. They include multiple heterogeneous groups, from GLAM professionals and scientific experts (e.g. conservators, curators and ‘front-of-house’ staff catering for visitors) to artists, volunteers, supporters, donors, friends, as well as other ‘surrounding’ culture-based or social groups that take an interest in the institution and its heritage collections and resources. In a commons-oriented ecology, all these groups shall be able to engage with the institution’s work (although through different degrees and modes of participation) and in several aspects; from the building of collections to co-curation, interpretation and public presentation. Ideally, as we will elaborate further later on, community members would be also involved in governance and management (e.g. directly or through representation in decision-making bodies and committees).

Overall, commons-oriented GLAMs cater for developing an institutional practice and organisation culture that ensures their services and assets remain accessible to their communities, audiences and beyond. This could be pursued through a range of strategies and tactics such as, eliminating economic barriers to access (e.g. devising an appropriate ticketing policy of free or low entrance fees, concessions etc.), providing space and resources to community groups to host their events, co-organising common projects, supporting local or under-privileged artists and professionals and through their content and programming, designing thematic exhibitions and events that bring out and expand neglected or conflictual aspects of urban history, give voice to the marginalised or allow for reflecting critically with contested heritage.

Thirdly, the porous and plural community is assembled through a number of self-regulated codifications (rules and norms/ informal and formal practices of preserving, reproducing, exhibiting, etc.) about the ways of managing and using the common resource. ‘Rules and norms’ represent here processes of institutional governance, such as strategic decision-making, but also day-to-day management arrangements that are developed to serve the vision and mission of the organisation, while securing its future viability and economic health. As implied earlier, it is crucial for GLAMs to be re-assembled in ways that will enable increased possibilities for museum staff and communities to assert control over the production and management of relevant resources. In doing so, the commons approach can bring to the fore a number of important social and collective values, such as inclusive democracy and solidarity, while also providing greater freedom and capacity to negotiate with contested heritage, colonial legacies and authoritative heritage narratives ( Silverman, 2011; Smith, 2006).

Conventional GLAMs (both public and independent non-profit institutions) are usually governed by a central committee, in the form of a board of directors, which takes all major decisions regarding strategy and management. Who participates or is represented in such committees and how porous are such decision-making bodies shall be considered as determining factors: (a) for management structure and organisational culture within a memory institution (both decisions themselves and the ways through which these decisions are taken determine and reproduce codes of conduct and values within an institution), (b) more broadly, for reproducing or transforming relations of power, ownership and control over processes for the creation, use of and access to heritage resources, narratives about the past and identity-making.

Since commons-oriented GLAMs shall be based upon (or at least incorporate to a certain degree) democratic practices and participatory mechanics that can sustain inclusion (and control), tuning collective processes, deliberating and selecting between different options as part of the organisation’s decision-making is of central importance. Therefore, commons-oriented GLAMs need to establish inclusive and horizontal governance arrangements, reflected also on their decision-making mechanisms. Favourable arrangements may include for example, general assemblies and extended boards that operate through open processes and have a diverse composition representing various actors (e.g. staff, volunteers, artists, members and users etc.). Such mode of operation would also accommodate current sectorial demands for ‘bottom-up’ heritage management (see among others, Beeksma & De Cesari, 2019; Chirikure *et al*., 2010; Pierroux *et al*. 2020; Stephens & Tiwari, 2015). With this in mind, our schema assigns ‘governance and management’ with a central, all-encompassing position in commons-oriented GLAMs ( Figure 1).

As depicted graphically, commons-oriented GLAMs need to be approached through a relational lens, where in practice, they allow their communities to use and combine various tangible and intangible resources, as described in our schema (i.e. labour, ideas, knowledge, digital tools and so on) to protect, re-produce and promote representations and interpretations of the past that are most relevant to the present in terms of realising collective benefit. Both, the community and the common resource are in a continuous state of mutual becoming when viewed as an assemblage; the community is territorialized (and its identity is consolidated) when it is starting to devise codifications over the use and management of the common resource, while the latter becomes significant and starts to hold transformative power when it is protected, reproduced and disseminated in certain ways, through codifications. At the same time these codifications define the social processes that make the cultural resource a commons; such as the degrees and levels of appropriation and access.

The resources are metabolised into ‘outputs’ in the form of exhibitions, cultural events, educational programmes and other channels of new knowledge in a feedback loop: in the commons ‘circuit’ they can serve as new resources/input for future work inside and outside the organisation. Thus, commons-oriented GLAMs perform similar tasks and generate knowledge and services comparable to that of typical GLAMs (in terms of output, they still serve the sectoral goals of heritage conservation/protection); the distinction is that this work is performed differently, introducing new processes to GLAMs’ institutional practice. This holds great potential for addressing the sector’s challenges: since it is done collaboratively, it is inherently more inclusionary (thus, potentially more socially relevant and representative; although also more susceptible to conflict), it is community-focused (thus, serving public benefit dynamically instead of prioritising some static ‘universal’ values) and it draws on an extended pool of talent, labour and ideas (thus, tackling understaffing/underfunding issues). In the next section, we elaborate further on commoning mechanics and specifics, highlighting some critical aspects of this ‘porous circuit’.

## What distinguishes ‘commons-oriented’ GLAMs: highlighting key principles

Organisational arrangements of GLAMs normally fall short in emerging or developing in ways that would allow their classification as commons. Yet, admittedly, our analysis so far discussed various commons-oriented practices that are not entirely unknown to the sector. In fact, some are already well-integrated, such as community-focused programmes and volunteering, whereas others are still largely unfulfilled but long desired, such as greater degrees of participation across institutional practice. Relevant ‘commons-like’ practices, even though fragmentary or casual, are not overall absent whereas in recent years, new digital tools and technologies have allowed the development of crowdsourcing, crowd-curation and co-creation projects, crowdfunding, and open-access digital collections into standard practices for a vast number of memory institutions in Europe. These practices resonate with a commoning culture and can ‘push’ small and medium-size institutions struggling with limited finances and audiences towards a more organised ‘commons’ direction.

At this primary conceptual stage, it is thus important to identify some key features that can serve as criteria for distinguishing commons-oriented GLAMs from traditional institutions. These criteria fall into 9 areas of interest: (i) legal/ownership status, (ii) decision-making, (iii) accessibility/engagement, (iv) labour, (v) relations with external actors, (vi) finance, (vii) content creation, (viii) sharing/distribution, (ix) open access and IPR.

### Legal/ownership status

The legal form and ownership status of a memory institution determines both its internal and external relations (e.g. with networks, community groups, market actors). We anticipate that legal/ownership capacities can either enable or prevent commoning practices to emerge. It is safe to say that in their vast majority, memory institutions in Europe are either state-owned or fall under the civil society/third sector category. Third-sector organisations are non-governmental (although they may still rely largely on public funding) and destined to serve citizens’ interests (e.g. as compared to shareholders’ interests of commercially-oriented businesses). The non-profit status prevents GLAMS from treating their outputs and products as ‘commodities’ and normally expected to discourage a ‘trade-off’ between profit generation and access (e.g. highly-priced memberships, reproduction fees etc.). Synergies with Social and Solidarity Economies can further trigger the cultivation and flourishing of commoning practices in GLAMs due to (a) their association with pre-fixed, horizontal decision-making processes (such as one member - one vote in the case of SSE initiatives and cooperatives) and (b) their commitment to openness and accessibility. We already have some successful examples from the GLAM sector, where such synergies have taken place in the form of community-based museums/ecomuseums, libraries and archives organised as associations, foundations, or charity organisations, or local firms operating as tourism, recreation and education/training co-operatives linked to a GLAM institution (see inter alia, Gonzalez *et al*., 2017; Gould, 2017; Iaione *et al*., 2022; Riva, 2017).

### Decision-making

As mentioned earlier, we consider commons-oriented GLAMs to adopt governance arrangements that are inclusive and horizontal; a condition which should also hold for decision-making mechanisms. In this direction, favourable arrangements include general assemblies and extended boards, which operate through open processes. Their composition shall also represent a diverse set of actors involved in GLAMs’ mission and work, such as ‘front-of-house’ and ‘back-of-house’ paid/volunteer workers, artists, audiences/users and heritage communities, namely communities of people who take public action to support, transmit and disseminate the values of cultural heritage that is held at memory institutions ( Council of Europe, 2005).

### Accessibility and engagement

As mentioned earlier, commons-oriented GLAMs should develop practices that ensure their resources, services and infrastructure are available for and accessible to their audiences, local and broader communities (e.g. through free entry to exhibitions, sharing of spaces, free distribution of educational materials). Moreover, efforts for engagement and participation that nurture collaboration (already present in the sector) allow GLAMs to serve as agents for social inclusion and empowerment, through representation, diversity, knowledge sharing and skills development (see inter alia Fleming, 2013; Klinenberg, 2018; Labadi, 2007; Simon, 2010). Overall, it is crucial for GLAMs to be re-assembled in ways that will enable increased possibilities for communities (e.g. ‘non-expert’ users, audiences, citizens) to work with GLAMs professionals sharing control over the production and management of relevant resources, in order to enact and create different kinds of knowledge through ‘doing heritage’ ( Vergunst & Graham, 2019). In turn, state authorities need to recognise the legitimacy of commons-oriented GLAMs to also present and interpret their versions of history and heritage, engaging with them in public dialogue for the benefit of culture, monuments and common good.

### Alternative modes of labour

Regarding labour, beyond the mainstream modes of labour (i.e. waged labour, service provider contracts etc.) and volunteering, which is already an established practice in the sector, commons-oriented GLAMs can also employ other alternative modes of labour, such as reciprocal and communal labour. Such alternative modes of labour are key components of the commoning praxis, framed by De Angelis (2017) as a ‘social labour flow pushed by needs, attracted by desires and oriented by sense horizon and aspirations’. Mobilised by and articulated around broader societal and political objectives, members of commons-oriented GLAMs can contribute to their everyday operation and reproduction and, subsequently, to their financial autonomy and sustainability and the creation of stable community relations through these modes of labour.

### Finance

GLAMs’ operation and reproduction is partly (alongside with labour) building on a variety of financing mechanisms and streams of income (membership/entrance fees, public funding, corporate funding/sponsorships, crowdfunding etc.). Among these, there are some financing streams that could operate as obstacles to the emergence of commoning practices, undermining the autonomy of GLAMs, as in the case of dependencies on public and private funding that often ‘translates’ into an increased power over governance and decision-making. Furthermore, own-generated income streams from user fees, visitor and education services could introduce limitations to openness and accessibility, when these financing streams are not treated and mobilised as a common pool resource but, instead, as an input that is accompanied by top-down hierarchies and specific modes of agency. As Gould (2017) has suggested, GLAMs produce goods and services with market value; a commons-oriented operation calls for mechanisms to prevent enclosures and assure commoners’ benefit (social/economic). For example, culture/heritage-related services (e.g. education, guided tours) could follow the principles of Social and Solidarity Economy, forming a line of defence against expansionist co-optation attempts by market forces.

### Content co-creation

Simon (2010) defines ‘co-creation’ in GLAMs as a process whereby communities and professionals co-set the project’s goals and work together throughout its implementation. Co-creation processes can be linked to several key functions of GLAMs, including research, inventorying, interpretation, cataloguing, communication and presentation to the public, in turn leading to the production of free and open-access goods and services, including exhibitions, guided tours, outreach programmes and education materials. GLAMs can further harness the new possibilities that are opened-up by digital commons to ‘peer-produce’ content ( Bauwens, 2009) through digital means and technologies at hand (e.g. interpretation of artefacts, anecdotal information, artistic work). As proposed by Manacorda (2016), treating the public as ‘a collaborator in an equal exchange’ during the curatorial process can cultivate a dynamic pedagogical habitat where ‘questions are asked and answers are constantly negotiated’ allowing for knowledge not merely to be gathered but to be actively produced. After all, to design ‘a museum of commons’, culture professionals shall not ‘design it to involve the public [but] to design it with the public’ (ibid 2016: 7, emphasis added). By incorporating users’ perspectives/needs in GLAMs programme and encouraging creativity and active participation, commons-oriented GLAMs can broaden the idea of openness beyond ‘open access’ towards an open culture that invites community contribution to the ‘core’ of GLAMs’ work ( Sanderhoff, 2014), such as curatorial and archivist.

### Knowledge sharing and distribution of outputs

As highlighted earlier, the main output of cultural/heritage commons is non-rival and shareable knowledge, both scientific and social. Science and arts knowledge can become open and accessible by harnessing new digital means, such as digital repositories of digitised objects and metadata, cloud-based infrastructure or open licensing ( Cousins, 2014; Edwards, 2015). This shared knowledge can form the basis for inviting current and future commoners to contribute to a polyphonic interpretation and signification of artworks and collections of GLAMs or for engaging in critical conversations regarding heritage that it is contested. In this light, knowledge ‘can be considered part of the resources, but also part of commoning, a product of social interaction and production by the various communities mobilised around the cultural resources, providing new meanings in their biography’ ( Lekakis, 2020b). Parallel with this, the production of social knowledge and civic skills, as a product of social interaction in a commons-oriented context, can be fed back and inform governance processes ( Klinenberg, 2018). The ways people interact with the resource and with each other are thus, output inextricably linked with a commons-oriented GLAM ( Madison *et al*., 2010).

While ‘internal’ processes of the GLAMs, namely the ways different components come together, enabling or posing barriers to the emergence of commoning practices is of key importance, it is also crucial to investigate the potentialities of the GLAMs to emerge as commons in the cultural sector, referring to both fulfilling broader societal goals and processes of institutionalisation involving commons-oriented GLAMs and other urban actors (e.g. local and regional governments, the civic society, the third sector, social movements, other modes of urban commons etc.). For example, the knowledge produced by GLAMs operating as commons can have positive spillover effects on the broader heritage and culture sector by informing professional practice for sharing, co-creating and co-curating the past, thus helping fulfil the ‘paradigm shift’ towards greater participation. In this vein, the ways outputs are shared and distributed should enable the maximum of positive spillover effects (e.g. cultural vibrancy, social cohesion, economic well-being) in the settings in which GLAMs are nested, but also to more remote audiences and communities through the employment of digital means.

### Open access and IPR

The commons’ premise necessitates wide and open access to resources at hand and assumes a feedback loop where a portion of the system’s output is used as future input. As prescribed by Europeana (2010), GLAMs need to defend creative artworks in the Public Domain (e.g. books, photographic archives, audiovisual materials) by waiving restrictions to their access, dissemination and adaptation so that society can re-use, re-interpret and re-produce these resources to create new ideas and works of art. For recent work that has not entered the Public Domain open license systems, such as Creative Commons (CC) can be used to allow for open access and distribution. CC licences are already popular across countless European cultural institutions, as they accommodate different levels of flexibility in terms of access, sharing and use, already popular across countless European cultural institutions ( Von Haller Grønbæk, 2014). Open access can lead to win-win outcomes for cultural institutions and their audiences already attested by sectorial experience ( Sanderhoff, 2014), as photographic collections, artwork imagery, digital materials and metadata can immediately gain more visibility when harnessing digital open distribution methods.

### Relations with external actors

GLAMs do not operate in the void but, instead, partake in networks and flows, which also extend beyond the cultural landscape. These ties and connections, along with their impact on GLAMs is of crucial importance, as they enable the emergence of both informal and formal streams of support that extend from financing and sharing tangible and intangible resources to the establishment of relations that attribute legitimacy or reinforce the scaling up of commoning practices, along with their institutionalisation. In this vein, the associations between GLAMs and actors/ networks that are driven by commons-oriented aspirations, motivations and logics push towards further implementation of commoning practices while, and most importantly, providing access to a diverse and extended pool of resources and support that, overall, can contribute to the augmentation of their collective capacities to be sustainable, resilient and, through the extended outreach, transformative. In this way, commons-oriented GLAMs can produce broader societal benefits through the spread of democratic values, place attachment, sense of belonging, solidarity, social cohesion, ultimately, nurturing a vibrant cultural sector that enriches the urban layout of cities.

Table 1 presents a synopsis of these commons-oriented practices for GLAMs. Obviously, these practices are not articulated in pre-fixed but in relational ways within a given organisation, leading to varied capacities and potentialities for operating as commons. This implies that each practice does not have some de facto ‘contribution’ to the making of a commons-oriented institution by itself, but rather the ways these practices are articulated and relate to each other are those which determine whether eventually, relationally, they will emerge together as empowering factors that create the conditions for a commons-oriented GLAM.

**Table 1.** Commons-oriented practices in the GLAM sector.

Area of interest	Commons-oriented practices
Legal and ownership status	Non-profit entities (associations, charities); Local co-operatives, SSE initiatives; Informal grassroots/citizen movements
Decision-making bodies and processes	General assembly, extended boards, working/operating groups, pop-up project-based teams; Inclusive, horizontal, representational, porous – rotated
Accessibility and community engagement	No or low tickets, concessions; Granting spaces/facilities/equipment (no charges); Diverse/representative programme and content; Support/inclusion of local and under-privileged artists/GLAMs professionals; Broad participation in management/governance by local communities/groups, ‘non-experts’, audiences, users
Labour	Waged and alternative modes (volunteer, reciprocal, communal)
Finance	Low dependence on corporate and state funding; Co-funding mechanisms (e.g. memberships, voluntary contributions)
Content creation	Co-set project goals and implementation; Participatory mechanisms for most aspects of GLAMs work (e.g. research, inventorying, interpretation, cataloguing/archiving, curation, communication, outreach, education etc.); Digital tools for peer-production
Knowledge sharing - distribution of outputs	Open and accessible knowledge across liberal arts (history, literature, creative arts etc.), through open-access physical archives and digital repositories, cloud-based infrastructure, open licensing etc.; Shareable social and professional knowledge
Open access and IPR	Resources create a feedback loop where a portion of the system’s output is used as future input; Open license systems, such as Creative Commons
Relations with external actors	Actors/networks driven by commons-oriented principles/mentality, within the GLAM sector and beyond (e.g. urban commons, SSE, social movements)

**Figure 2.**
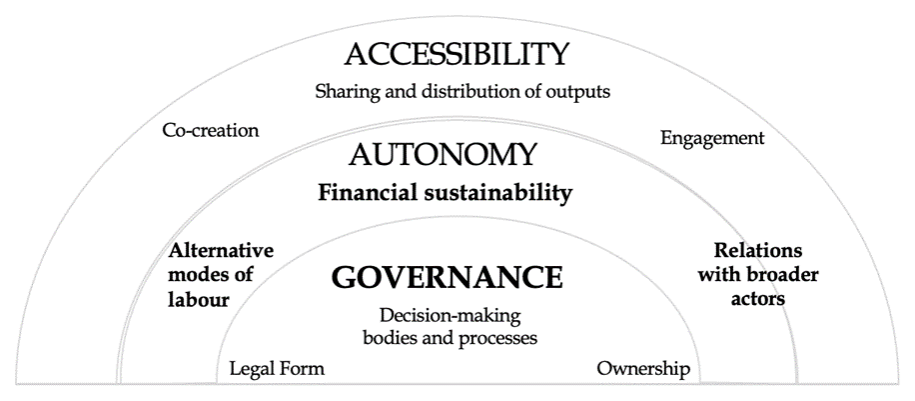
Key principles of commons-oriented GLAMs.

Still, it would be useful to organise these practices and relations hierarchically, as some of them lie at the core of commons-oriented GLAMs, paving the way for more ‘peripheral’ and ‘ancillary’, yet crucial practices to emerge. Based on our analysis so far, we identify three key principles that determine GLAMs’ potential to operate as commons: (a) governance, (b) autonomy and (c) accessibility ( Figure 2). The proposed hierarchy is useful for preventing us from understanding commoning practices as a ‘checklist’, comprising equally influential, independent sets of practices. Instead, we need to treat these elements as intercommunicating and relational practices that, through their interactions, are mutually reinforced and jointly contribute towards the realisation of each principle.

As shown in Figure 2, there are certain practices that relate directly to one of the aforementioned principles (e.g., decision making practices to governance), and others which have an extended scope across different principles. One case in point is labour, which is relevant to all sets of practices (and criteria) described here. Similar to commons in other sectors, commons-oriented GLAMs may draw on and develop resources and goods that are diverse and heterogeneous (e.g. repositories, registries, collections, exhibitions, works of art) but ‘their common denominator is that they involve shared resources which are governed, produced and distributed through collective participation’ ( Kioupkiolis, 2022). Thus, on principle, commons-oriented GLAMs would feature cultural goods which are collectively produced, shared and used ( Kioupkiolis, 2022). What would mark off these cultural/heritage goods as ‘commons’ is their ‘near-egalitarian mode of self-organising their production, management and distribution’ (ibid). Following that, commons-driven governance arrangements lay the ground for commons-oriented GLAMs, representing the key principle and a prerequisite for the emergence of any further commoning practices. A memory institution cannot be considered as commons or commons-oriented in the absence of relevant governance arrangements (e.g. horizontal, democratic, inclusive), ideally accompanied by an appropriate legal and ownership status (e.g. community-based, non-profit, cooperative).

Admittedly, a transition from the current canon of centralised heritage management to a commons governance would require devolving management authority from the top to the bottom, thus challenging long-held political, legal, economic and attitudinal realities across the sector. At organisational level, well-established GLAMs would be most likely reluctant to share their materials and knowledge in a commons setting, where they will have limited control and authority ( Marttila & Botero, 2017). Thus, a commoning ethos necessitates a negotiation of the professionals and experts’ role as the primary custodians of culture/heritage resources (e.g. collections, monuments, archives) so that commoners can be granted legitimate authority to act upon the management and governing of GLAMs. Given that ‘community actors need to be proactively involved in design and co-management’ ( Iaione *et al.*, 2022), they need to work closely with GLAM professionals with the view to build a common ‘language’, channels for reciprocal knowledge transfer and mutual understanding so that the roles and areas of contribution can be co-decided and re-coded for each party. From a pragmatic point of view, GLAMs that are missioned to protect national ‘treasures’ and present dominant versions of the past have little flexibility to accommodate commons practices as compared to institutions specializing for instance, in local histories, subcultures, rural heritage or contemporary archaeology.

Moreover, decision-making within a GLAMs ecosystem extends to interpretation and contestation of diverse and often antagonistic values, perspectives and narratives. Here, we need to pay attention to the boundaries that distinguish genuine efforts towards a commons paradigm from ‘commons-inspired’ participatory projects, or even ‘commons-washing’ attempts. We shall view heritage itself as a territory and a process of negotiating with the past. In the realm of interpretation, an ‘opening’ to marginalised voices which shall not be viewed as a mechanistic process to reject official narratives and celebrate non-elitist memories. Rather, endeavours need to focus on constructing critical and timely accounts about the past in the present. Since cultural/heritage resources are dynamic, their interpretation and management needs to be revisited systematically in order to capture the values that are invested in them at a given time and place, engaging commoners in ‘critical memory’ projects to deconstruct hegemonic (and also, popular reactionary) discourses, to collectively choose the ones that are indeed ‘progressive and emancipatory’ ( Gonzalez-Ruibal, 2017).

Another critical set of practices is those that build towards the second key principle of autonomy from state and market actors, by limiting dependencies on external actors that antagonise GLAMs’ ‘commoners’ for power and control. Financial autonomy in particular is crucial, as in many cases experience shows that relevant dependencies can lead to the overwhelming agency on behalf of state and market actors, imposed through top-down and closed modes of governance and decision-making. The third principle concerns accessibility, materialised and secured by commoning practices and ethos. Accessibility here concerns the modes of engagement with and the role of communities and users/audiences in benefiting from relevant resources, content creation and the sharing/ distribution of knowledge and outputs. Co-creation and engagement practices, knowledge-production, exchange and transfer processes are neither intended to undermine professionalism nor substitute expert workers with ‘amateur’ creators but rather, replace monologue with dynamic multi-vocal conversations ( Tapscott & Williams, 2008), which would enable the use and management of collections by groups of actors who are the collections’ communities whilst being accountable to a wider public ( Graham, 2017), nurturing a critical approach to the past - especially when dealing with contested heritage.

Towards exploring the application, flexibility and adaptability of the framework presented in this paper, a series of limitations can be brought out. First, within the current GLAM – and broader cultural - landscape in Europe, only a limited segment of existing initiatives and organisations fulfill the majority of the criteria laid out and discussed here. These mostly comprise small-scale, informal, grassroots and – often – politically motivated entities in urban areas. The cases of Teatro Valle in Rome ( Bailey & Marcucci, 2014), the Schwules museum (LGBTQ+ history and culture museum) in Berlin and other community museums, neighbourhood libraries, mass observation/ oral history ( Summerfield, 1985) initiatives and networks that produce historical archives are indicative of the aforementioned fragment. These small and community-led initiatives usually enter the schema in Figure 2 from the bottom and run through the three levels/principles, employing commoning practices from the everyday management and strategic governance of their organisations, up to the levels of securing autonomy and offering wide accessibility of their archives, museum collections etc, and thus can be regarded as commons per se. However, and as noted above, the majority of GLAMs cannot be considered as commons per se organisations; although we find a lot of examples where GLAMs employ commoning practices, especially at the level of sharing their outputs widely and for free. But these organisations, while entering the schema from the top, usually stay at that level of accessibility and do not enter the next two levels of commoning practices. This is true for large-scale organisations - most often highly dependent upon state funding or building on for-profit logics; for these organisations it would take a fundamental shift of character to comply with several of the criteria discussed earlier. Towards such a development, besides their dependencies on state and market actors, their closed and hierarchical governance arrangements, the difficulties towards coming up with alternatives for covering high operational costs, along with integral exclusionary aspects of their function (may them be the lack of open access large institutions with an entrepreneurial logic or the exclusion of counter-hegemonic narratives in state-related ones) pose crucial and most likely formidable barriers towards adapting several commons-oriented practices. 

Nevertheless, we argue that the commoning practices and principles discussed here could i) inspire existing GLAM entities that are situated between the informal, community-led/ grassroots and the large, well-established organisations and ii) pave the way for the emergence of relevant initiatives deriving outside narrow classifications of cultural institutions, namely urban commons, social movements and civic society networks, initiatives of the Social and Solidarity Economy who want to be mobilised in the cultural landscape or expand their repertoires of actions, opening up to and creating ties with local communities through the in-common, co-production of cultural infrastructure and content and, thus, broader common understandings of local history and collective memory, iii) challenge GLAMs to reconsider the relationship between practices of co-creation and audience development with providing more institutional space and devolving some of their power to their users/audiences, by employing more commoning practices in the level of governance. Again, certain questions and limitations arise, as the proposed model can be adapted more easily by small-scale initiatives that build upon existing networks and social ties (e.g. for the provision of non-waged labour) which also include actors who are familiar with horizontal governance processes. 

Furthermore, the prospects for the effective implementation of the commons-oriented GLAMs framework, alongside the possibilities for the expansion of pertinent initiatives and networks, are influenced by the presence or absence of supportive policy and legal/institutional contexts, particularly at the municipal level. Environments conducive to the advancement of commons-state institutions can be identified in municipalities that have embraced the paradigm shift in urban governance known as new municipalism, characterized by collaborative partnerships between local authorities, social movements, civil society, and urban commons. Notably, the formation of relevant institutions and networks does not result from unilateral actions by local governments, but rather from dynamic, continually negotiated processes that involve both collaboration and conflict, aimed at reorganizing aspects of urban production, social reproduction, and public infrastructure in alignment with principles of democracy, ecology, and egalitarianism ( Thompson, 2020).

Bianchi (2023: 2129) contends that Commons-state institutions are contingent upon the synthesis of interpretations of self-government and non-appropriability, primarily by various segments of civil society and state actors. In essence, they represent negotiated institutional arrangements. Additionally, Kioupkiolis (2020a) underscores the significance of social movement practices and legal mediation in facilitating institutional shifts in Italian cities toward fostering commons-state alliances, beyond mere political will on the part of local authorities. With an appropriate legal and administrative framework, such as the Bologna/Labsus model (see Kioupkiolis, 2020b), local authorities can bolster urban commons by providing resources and infrastructure that can be integrated into commons-based social systems. Notable examples of successful initiatives within this context include Teatro Valle in Rome and L’Asilo in Naples, which exemplify the potential of commons arrangements in cultural production.

## Concluding remarks and future research

Both prolonged (e.g., the neoliberal and entrepreneurial shifts in cultural and urban policy frameworks and governance) and recent (e.g., the COVID-19 outbreak) developments have raised crucial questions concerning the sustainability and the broader societal role of cultural and memory institutions. Acknowledging that the commonplace ‘top-down’ model of governance has often limited capacity to address effectively the current challenges of the sector, researchers have often seen the commons paradigm as promising a collective resolution to the management of shared resources for the production of goods and services that deliver shared benefits. However, the review of the available literature suggests that GLAMs as commons lie in potential, yet to be realised and take a full form in both theory and practice. Commons-oriented management of cultural/heritage resources (tangible, intangible and digital) is a new and largely uncharted field. The commons can help cultivate a ‘new culture’ in the sector; a culture of ‘co-creation, sharing and pooling productive knowledge and other resources’ ( Kioupkiolis, 2022). Apart from technology, grassroots initiatives and civil society groups can be catalysts for the transition to a commons-oriented GLAM sector, supported by law and the economy.

This paper attempted to situate the GLAM sector within the Commons theoretical framework, taking into account the specificities and challenges of the GLAMs (as a mere homogenous sector) and the variegated approaches of the commons literature. What we propose, is a new conceptualisation of GLAMs as commons where various elements from the commons literature inform a new conceptual framework where commons-oriented GLAMs can operate. In this direction, we provide a set of criteria, each referring to different aspects of GLAMs’ operation, arguing that their articulation can contribute to the emergence of sustainable, inclusive and democratic memory institutions. We believe that that new framework can offer a device to all those communities that are assembled through a collective interest over a particular historic past, and they are willing to preserve and transform it via a commons-oriented management structure and ethos.

By proposing commons as a paradigm for GLAMs, we wish to inspire future research into the potentialities of commoning practices to provide sustainable solutions for a financially- struggling sector that, in principle, serves public interest. While neoliberal politics are increasingly colonising public affairs, we view commons as a viable avenue to securing financial resilience by passing market logic, maintaining commitment to the sector’s society-oriented vision. Future research needs to explore further how commons-based practices translate practically in the GLAM sector, using our conceptual framework as an analytical tool for interpreting related empirical data.

## Ethics and consent

Ethical approval and consent were not required.

## Data Availability

No data are associated with this article.

## Footnotes

^1^ See http://oralhistorygroups.gr/ [in Greek]
