# Supplementary material for: Rethinking GLAMs as commons: a conceptual framework
Source: Open Res Eur. 2024 Jul 9;3:157. Originally published 2023 Sep 21. [Version 2] doi: 10.12688/openreseurope.16473.2 (PMC11267147; doi:10.12688/openreseurope.16473.2)
Supplement: Peer review history

# Reviewer report: Reviewer response for version 2

**DOI:** 10.21956/openreseurope.19534.r41999

**Published:** 2024-07-23

**Authors:** Alice Borchi

**Affiliations:** 1 University of Leeds, Leeds, England, UK

My comments have been addressed in a a detailed way; I've just spotted a minor typo - it's Teatro Valle, not Valley. I am happy to approve this version of the article.

If any, are all the source data and materials underlying the results available?

Yes

Is the work original in terms of material and argument?

Yes

Is the work clearly and cogently presented?

Partly

Is the argument persuasive and supported by evidence?

Yes

Does the research article contribute to the cultural, historical, social understanding of the field?

Yes

Does it sufficiently engage with relevant methodologies and secondary literature on the topic?

Yes

Reviewer Expertise:

My research expertise is in the area of cultural policy, cultural values and the cultural commons. My work has been featured on relevant international academic journals and conferences, and I am currently leading an interdisciplinary seminar series on the contemporary understandings of the idea of the commons across different academic disciplines.

I confirm that I have read this submission and believe that I have an appropriate level of expertise to confirm that it is of an acceptable scientific standard.

# Reviewer report: Reviewer response for version 2

**DOI:** 10.21956/openreseurope.19534.r42000

**Published:** 2024-07-17

**Authors:** Peter Gould

**Affiliations:** 1 Penn Cultural Heritage Center, University of Pennsylvania Museum, Philadelphia, PA, USA; 2 Indiana University Bloomington, Bloomington, Indiana, USA

The author's  were extremely thoughtful in their handling of the issues and I think this is a worthwhile paper.

If any, are all the source data and materials underlying the results available?

No source data required

Is the work original in terms of material and argument?

Yes

Is the work clearly and cogently presented?

Yes

Is the argument persuasive and supported by evidence?

Partly

Does the research article contribute to the cultural, historical, social understanding of the field?

Yes

Does it sufficiently engage with relevant methodologies and secondary literature on the topic?

Yes

Reviewer Expertise:

I work on the economics of cultural heritage and have published frequently on issues of the commons framework as it applies to heritage, including papers cited by the authors in this article. I am chair of the University of Pennsylvania Museum Board of Advisors, have chaired the boards of two other substantial cultural institutions, and served on the boards of several more.

I confirm that I have read this submission and believe that I have an appropriate level of expertise to confirm that it is of an acceptable scientific standard.

# Reviewer report: Reviewer response for version 1

**DOI:** 10.21956/openreseurope.17782.r35283

**Published:** 2023-11-08

**Authors:** Alice Borchi

**Affiliations:** 1 University of Leeds, Leeds, England, UK

The authors present a convincing and well-designed framework that presents opportunities for further studies and practical application both from the perspective of both cultural policy and management. The paper engages convincingly with existing literature on the commons, and the interdisciplinary perspective adopted by the authors proves to be particularly effective for the purpose of their paper. This is a welcome and rigorous contribution to the study of the cultural commons. 

 The necessity of rethinking the management of GLAMS is well contextualized. However, considering that the paper focuses on memory institutions, it is necessary for the authors to discuss the notion “contested heritage” (Silverman, 2010). De-colonial, anti-racist and repatriation movements have significantly changed heritage discourse in Europe; furthermore, heritage has become an arena for the discussion of controversial historical figures, resulting with protests and, in some instances, the removal of statues or other heritage sites. This contextual element is important for the paper for two reasons: first, it is necessary to discuss how the governance mechanisms proposed by the authors can address these issues and possibly generate democratic pathways to the management of contested heritage. Secondly, the notion of contested heritage raises useful questions about the relationship between “heritage” and “community”: whose heritage? Whose memory? Whose values? Whose community?  The authors state: “We believe that that new framework can offer a device to all those communities that are assembled through a collective interest over a particular historic past”: are there any examples/explanations in the existing literature of how these communities are formed, and how they operate? The authors reference Bailey and Marcucci 2014 to illustrate this point, but the case discussed by Bailey and Marcucci is a very particular one, Teatro Valle: this theatre was occupied by activists and professionals of the cultural sector, who were evicted from the theatre in 2014, as the local council did not recognize nor the occupation nor the management of the theatre by the commoners as legitimate. This source illustrate how the sustainability of the commons is endangered by a lack of legal recognition of the commons and by the hostility of local authorities towards forms of collective management that operate autonomously and in opposition to existing policies. All these problems are useful in thinking about the potential of commons-oriented practices in the context of GLAMS in addressing issues of inequality in the context of GLAMS. 

 The issue of “community” could be further examined in the analysis of the existing literature on the cultural commons; in particular, I think that the work of Merima Bruncevic on law, arts and the commons could be an interesting source for the authors to examine. 

 From a conceptual standpoint, more clarity is required in connection to the following questions: 

1. How flexible is the framework presented by the authors? In order to self-describe as a “commons-oriented GLAM”, does an organization need to satisfy all the criteria contained in the framework, or is this an adaptable model? More broadly, this question refers to the extent to which the framework is to be understood as normative for the sector. 
2. What are the challenges that the authors expect GLAMs to face when engaging with the framework in terms of scale, expertise and resources? Which GLAMs might find it easier to implement?
3. From a policy perspective, the legal recognition of the status of “commons” tends to vary significantly across Europe (and even across different cities in the same country, as it is the case in Italy). How does the existence, or the lack thereof, of policy on the commons affect the implementation of the framework proposed in this paper? Does the framework offer any opportunity to policymakers to engage with this issue? 

That said, I congratulate the authors on their rigorous work; the model they propose is clear and well structured, and directly addresses some of the major challenges of the contemporary cultural sector.

If any, are all the source data and materials underlying the results available?

Yes

Is the work original in terms of material and argument?

Yes

Is the work clearly and cogently presented?

Partly

Is the argument persuasive and supported by evidence?

Yes

Does the research article contribute to the cultural, historical, social understanding of the field?

Yes

Does it sufficiently engage with relevant methodologies and secondary literature on the topic?

Yes

Reviewer Expertise:

My research expertise is in the area of cultural policy, cultural values and the cultural commons. My work has been featured on relevant international academic journals and conferences, and I am currently leading an interdisciplinary seminar series on the contemporary understandings of the idea of the commons across different academic disciplines.

I confirm that I have read this submission and believe that I have an appropriate level of expertise to confirm that it is of an acceptable scientific standard, however I have significant reservations, as outlined above.

## References

[1] : Contested cultural heritage: religion, nationalism, erasure, and exclusion in a global world. *Springer Science & Business Media.* .2010;

[2] : Fixing the shadows–Access to art and the legal concept of the cultural commons. (Doctoral Theses). *University of Gothenburg* .2014;

[3] : Law, art and the commons. *Routledge* .2017;

# Response

**Published:** 2024-06-13

**Authors:** Dimitrios PETTAS

Dear Prof. Borchi, Thank you very much for your feedback. In what follows, we explain how we adressed your comments in the new version of our paper:

**Comment 1: **The necessity of rethinking the management of GLAMS is well contextualized. However, considering that the paper focuses on memory institutions, it is necessary for the authors to discuss the notion “contested heritage” (Silverman, 2010). De-colonial, anti-racist and repatriation movements have significantly changed heritage discourse in Europe; furthermore, heritage has become an arena for the discussion of controversial historical figures, resulting with protests and, in some instances, the removal of statues or other heritage sites. This contextual element is important for the paper for two reasons: first, it is necessary to discuss how the governance mechanisms proposed by the authors can address these issues and possibly generate democratic pathways to the management of contested heritage. Secondly, the notion of contested heritage raises useful questions about the relationship between “heritage” and “community”: whose heritage? Whose memory? Whose values? Whose community?  The authors state: “We believe that that new framework can offer a device to all those communities that are assembled through a collective interest over a particular historic past”: are there any examples/explanations in the existing literature of how these communities are formed, and how they operate? The authors reference Bailey and Marcucci 2014 to illustrate this point, but the case discussed by Bailey and Marcucci is a very particular one, Teatro Valle: this theatre was occupied by activists and professionals of the cultural sector, who were evicted from the theatre in 2014, as the local council did not recognize nor the occupation nor the management of the theatre by the commoners as legitimate. This source illustrate how the sustainability of the commons is endangered by a lack of legal recognition of the commons and by the hostility of local authorities towards forms of collective management that operate autonomously and in opposition to existing policies. All these problems are useful in thinking about the potential of commons-oriented practices in the context of GLAMS in addressing issues of inequality in the context of GLAMS.

**Response: **We are extremely grateful to you for reading our paper and providing us with your insightful comments. Firstly, we definitely agree that commons-oriented GLAMs need to engage with current heritage debates and with contested heritage. The latter is now more eloquently expressed;

- on page 6 of the revised manuscript, where we argue that commons-oriented GLAMs need to pursue greater access also through ‘their content and programming, designing thematic exhibitions and events that bring out neglected or conflictual aspects of urban history, give voice to the marginalised or allow for reflecting critically with contested heritage’.
- on pages 6 and 7, where we suggest that ‘the commons approach can bring to the fore a number of important social and collective values, such as inclusive democracy and solidarity, while also providing greater freedom and capacity to negotiate with contested heritage, colonial legacies and authoritative heritage narratives (Smith 2006; Silverman 2010)’.

Regarding democratic pathways to the management of contested heritage, we argue that in commons-oriented GLAMs heritage shall be seen as a territory for critically reflecting and negotiating with the past. As we explain on page 11: ‘In the realm of interpretation, an ‘opening’ to marginalised voices shall not be viewed as a mechanistic process to reject official narratives and celebrate non-elitist memories.  Rather, endeavours need to focus on constructing critical and timely accounts about the past in the present. Since cultural/heritage resources are dynamic, their interpretation and management needs to be revisited systematically in order to capture the values that are invested in them at a given time and place, engaging commoners in ‘critical memory’ projects to deconstruct hegemonic (and also, popular reactionary) discourses, to collectively choose the ones that are indeed ‘progressive and emancipatory’ (Gonzalez-Ruibal, 2017)’ Furthermore, the ‘whose heritage/whose community’ questions were also among our concerns while working on this framework. Considering the limited empirical evidence at hand, it is perhaps more safe to say that community formation processes are context-specific and idiosyncratic, so it would be hard to present a solid explanation about their operation. What we observe, and now mention in the revised manuscript, is that commons-oriented projects in the sector could emerge as a reaction to a current situation/problem (as in the case of Teatro Valley) or they may take a more proactive approach, e.g. as in citizen groups doing oral history. Please see our amendments on page 4: ‘In this light, heritage commons have been envisioned as dynamic and porous systems that protect the resources at hand while catering for the common benefit of involved communities (Lekakis, 2020). In such systems, cultural heritage can be understood as a resource that is compiled collectively, as part of the work performed by memory institutions operating in the GLAM sector, to promote greater representation and more democratic/multivocal discourses about the past. In line with other commons, this ‘collective production’ of cultural/heritage commons can bring together a community of commoners (Bailey and Marcucci, 2014). But, as scholarly work has emphasised, ‘communities’ (geographic, virtual or imaginative) are not always ‘community-like’ but rather incohesive assemblages of people with divergent interests (Waterton and Smith, 2010). Thus, communities with interest in the cultural/heritage resource shall be regarded as plural and geographically-unbound, and they may include culture professionals, art workers, heritage researchers, local residents, patrons and many others (Bailey and Marcucci, 2014). These communities are likely to be formed dynamically, reacting against the neglect or even hostility of public authorities (e.g. Teatro Valley in Italy; Bailey and Marcucci, 2014), or they may take a proactive approach to heritage-making (e.g. grassroots oral history projects, such as the ‘Oral History Groups’ in Greece’). We were also inspired by your observation that in the case of Theatro Valley ‘the local council did not recognize nor the occupation nor the management of the theatre by the commoners as legitimate’. With this in mind, we added a comment to our ‘principles’ section, page 8, saying that: ‘state authorities need to recognise the legitimacy of commons-oriented GLAMs to also present and interpret their versions of history and heritage, engaging with them in public dialogue for the benefit of culture, monuments and common good.’

**Comment 2: **The issue of “community” could be further examined in the analysis of the existing literature on the cultural commons; in particular, I think that the work of Merima Bruncevic on law, arts and the commons could be an interesting source for the authors to examine.

**Response: **Thank you for the insightful comments on the contested nature of (each and every) community and for the really interesting reference. We have included a more explicit theorisation of the contested nature of community, making reference to the notions of “agonistic pluralism” and “agonistic commons” at the end of the literature review, on pg. 4 ‘More fundamentally, and beyond the relations of communities to other, ‘external’, actors, as Bruncevic (2018, p. 143) argues in her analysis of the fluid and contested nature of community and property of cultural artefacts “one of the more significant problems with the concept of community is that it is often presupposed as homogeneous or at least as an, at any given point, definable group”. As, however, heterogeneity of actors and potential divergence of interests are characteristic of contemporary complex communities, conflict within the community over what may be considered cultural resources and how these may be optimally managed becomes a central issue to be addressed through collective commoning practices. Recognising that divergence and adversarious positions are inherent in communities, we may conceive of “agonistic pluralism” (Mouffe, 1999, 2007) as an essential element of community building, and recognise cultural commons as “agonistic commons” (Kioupkiolis, 2019), which could “nurture bonds of mutual recognition and coalescence among different parties who remain rivals along certain dimensions, making more space for plural freedom under conditions of dissent and division” (Kioupkiolis, 2019, p. 28). It is not, therefore, the existence of divergence and adversariality per se which is antithetical to the notion and practice of community, or to the practices of commoning devised by a community, but the management of such divergence in ways which will not presuppose the homogeneity or even uniformity of participating actors and entities. This brings to the fore the importance of institutions and processes of governance.’ Additionally, the ‘whose heritage/whose community’ questions which are relevant to the heterogeneous nature of communities and corresponding claims of rights over cultural goods and cultural resources were addressed in the manner outlined above.  

**Comment 3:** How flexible is the framework presented by the authors? In order to self-describe as a “commons-oriented GLAM”, does an organization need to satisfy all the criteria contained in the framework, or is this an adaptable model? More broadly, this question refers to the extent to which the framework is to be understood as normative for the sector/ What are the challenges that the authors expect GLAMs to face when engaging with the framework in terms of scale, expertise and resources? Which GLAMs might find it easier to implement? **Response: **We included two paragraphs right after the presentation of the framework and before the conclusions, discussing in detail issues of application, flexibility and adaptability of the framework presented in the paper.  

**Comment 4: ** From a policy perspective, the legal recognition of the status of “commons” tends to vary significantly across Europe (and even across different cities in the same country, as it is the case in Italy). How does the existence, or the lack thereof, of policy on the commons affect the implementation of the framework proposed in this paper? Does the framework offer any opportunity to policymakers to engage with this issue?

**Response:** We included two paragraphs on page 12, discussing the role of policy contexts and examples of successful partnerships between local authorities and cultural commons initiatives.  

**Comment 5:** Useful references: Silverman H. ed: Contested cultural heritage: religion, nationalism, erasure, and exclusion in a global world. Springer Science & Business Media. 2010/ Bruncevic M: Fixing the shadows–Access to art and the legal concept of the cultural commons. (Doctoral Theses). University of Gothenburg. 2014/ Bruncevic M: Law, art and the commons. Routledge. 2017.

**Response: **We thank you very much for providing us with these references. Please note that we now cite Silverman in our analysis of contested heritage. We have also included the work of Gonzalez-Ruibal (2017) which, similarly to Silverman, is more related to cultural heritage and heritage conflict, as well as Bruncevic (2017) on the contested nature of communities/rightful owners of cultural artifacts.

# Reviewer report: Reviewer response for version 1

**DOI:** 10.21956/openreseurope.17782.r35281

**Published:** 2023-10-11

**Authors:** Peter Gould

**Affiliations:** 1 Penn Cultural Heritage Center, University of Pennsylvania Museum, Philadelphia, PA, USA; 2 Indiana University Bloomington, Bloomington, Indiana, USA

The paper presents a cogent and comprehensive review of the relevant literature and presents an argument that contributes to debates in the field.  There is a great deal to praise in its structure, content and presentation. The authors clearly are scholars of the issues they are assessing.

 At the heart of this paper is the argument that so-called GLAMs can be conceptualized as a commons and that commons governance ideas may help the GLAMs adapt to contemporary demands on “memory institutions” to address local concerns.  I wholeheartedly agree that the GLAM sector globally is, and should be, undergoing a shift in focus from universal conceptions of cultural value to reflect both the contentious history underlying collections of items, whose origin stories are rooted in colonial or other exploitative endeavours in past centuries, and the distance of most GLAMs from the interests, needs and priorities of their adjacent communities.

 As the authors correctly note within the paper, I and others have in the past been skeptical of applying commons concepts to the heritage sector.  While I think the authors fairly present this critique, they are nonetheless confident in asserting that “it is clear that GLAMS fall into the category of new commons” (p 6).  This leads them to a conceptual framework that characterizes a commons-based GLAM as one that is or can be “a commons’ resource, a self-governing community, and a set of self-legislated rules and norms” (p6). This positioning reflects a tendency among scholars analysing heritage through a commons lens to simultaneously overemphasize the potential of GLAMs (and other heritage resources) to be governed as commons while, at the same moment, to underweight the governance considerations that make this highly unlikely to be true.

 At bottom, and I write here as a board member of museums and other cultural institutions as well as a scholar of Ostrom’s work, this is the point on which I part company with the authors and hope they will address the issues I raise substantively in a revision. 

 First, I believe they need to differentiate among types and scales of GLAM institutions. For example, I can readily accept their arguments if applied to community museums with low overheads and a mission focused on local community needs (such as one finds in many emerging countries in the Asia, Africa and Latin America).  These exist at small scale around the world and likely can (and should) be governed from a commons standpoint of local engagement, control and financing in which the critical rules are devised by locals for the benefit of locals. These are institutions, however, with limited audiences and financial needs.  I also could extend their model, with adjustments, to institutions that may be owned and funded by governments, but only by governments that are tolerant of innovative uses of collections and spaces to serve local community needs.  Nina Simon’s vision for the “Participatory Museum,” based on her experience at the city-owned by autonomous museum in Santa Cruz, California, illustrates many of these principles. Hers, however, was a free-entry museum in a very “liberal” community.  Many museums in the US and abroad are experimenting with community-curated exhibits and projects, but that is not the same thing as being community-driven.  Indeed, the larger and more prominent the GLAM enterprise, the more that governance realities intrude on an idealized version of how a “commons-oriented” GLAM would work.

 Second, and related,, in the case of large institutions (think the major Universal Museums, civically owned museums, libraries and so forth), the stakeholders engaged in these institutions are numerous (government owners, often separate government funders, philanthropic donors, professionalized staff members, sophisticated administrators, lenders and other financial overseers).  That complexity essentially makes the idealized notion of community-led, commons-oriented governance impossible.  Participatory measures that are taken may be token, and obstacles to community control impossible to surmount, especially where budgets are measured in millions of dollars or the equivalent. Such institutions, realistically, are unlikely to be “re-assembled in ways that will enable increased possibilities for museum staff and communities to assert control” (p7). Indeed, in many cases the professional staffs themselves would be opposed to delegating curation and presentation to non-“expert” community members.

 The authors suggest solutions, such as the representation of community members on governing boards or management and that rules and norms be assembled in a self-regulated manner. While the former is possible, it hardly emulates the sorts of self-organization and self-regulation envisioned in the commons literature. The latter, I would argue is exceedingly unlikely outside of small, local contexts. Legal ownership, fiduciary responsibility of directors, labour relations, and access all militate against a radical commoning of larger-scale and complex GLAM governance. It is not enough to say these things must change, as the authors do. They need to confront the reality that these changes fly in the face of substantial political, legal, economic and attitudinal realities. Not the least of these is that larger scale GLAMs require enormous amounts of money to operate, entitling the providers of such money (governments or philanthropists) almost insurmountable leverage over the conduct of the GLAM institution.

 I also am concerned that the authors have not acknowledged the contestation within communities themselves. Often “communities” based on geographic proximity are highly discordant in their values, aims and interests. One consequence of an idealized view of communities is that it tends to produce an expectation that “community generated” content will be more inclusive of differences and expressive of tolerance. There are, in fact, no guarantees of this outcome. Communities around the world are notoriously not homogeneous, but it is commonplace in scholarly literature to act as if they are.  Ostrom’s work i demonstrates the need for communities to resolve such differences and admits of the difficulty of doing so as commons institutions increase in scale.

 The authors identify three “key principles that determine GLAMs’ potential to operate as commons: (a) governance, (b) autonomy and (c) accessibility” (p10). My essential recommendation for this paper is that the authors ponder the implications of the arguments I am presenting and either offer a more nuanced evaluation of how the realities of scale, ownership/management structures, and financial dependence require them to modify their arguments *and/or* suggest how, in the context of real-world GLAM operations, they could feasibly adapt larger-scale GLAMs to overcome the limitations in governance, autonomy and accessibility that interfere with their vision of a commons-oriented GLAM sector.

 Let me emphasize that the matter these authors are exploring is of the utmost importance, and they are clearly equipped to make substantive contributions to advancing the GLAM sector in the ways they wish.  Perhaps, however, they stop too soon. I would hope that they have ideas for solutions to the issues I raise. At a minimum, they need to acknowledge these complexities more clearly and pose as future research priorities the identification of viable alternatives to contemporary realities.

If any, are all the source data and materials underlying the results available?

No source data required

Is the work original in terms of material and argument?

Yes

Is the work clearly and cogently presented?

Yes

Is the argument persuasive and supported by evidence?

Partly

Does the research article contribute to the cultural, historical, social understanding of the field?

Yes

Does it sufficiently engage with relevant methodologies and secondary literature on the topic?

Yes

Reviewer Expertise:

I work on the economics of cultural heritage and have published frequently on issues of the commons framework as it applies to heritage, including papers cited by the authors in this article. I am chair of the University of Pennsylvania Museum Board of Advisors, have chaired the boards of two other substantial cultural institutions, and served on the boards of several more.

I confirm that I have read this submission and believe that I have an appropriate level of expertise to confirm that it is of an acceptable scientific standard, however I have significant reservations, as outlined above.

# Response

**Published:** 2024-06-13

**Authors:** Dimitrios PETTAS

Dear Prof. Gould, Thank you very much for your feedback. In what follows, we explain how we adressed your comments in the new version of our paper:

**Comment 1: **As the authors correctly note within the paper, I and others have in the past been skeptical of applying commons concepts to the heritage sector.  While I think the authors fairly present this critique, they are nonetheless confident in asserting that “it is clear that GLAMS fall into the category of new commons” (p 6).  This leads them to a conceptual framework that characterizes a commons-based GLAM as one that is or can be “a commons’ resource, a self-governing community, and a set of self-legislated rules and norms” (p6). This positioning reflects a tendency among scholars analysing heritage through a commons lens to simultaneously overemphasize the potential of GLAMs (and other heritage resources) to be governed as commons while, at the same moment, to underweight the governance considerations that make this highly unlikely to be true […] First, I believe they need to differentiate among types and scales of GLAM institutions. For example, I can readily accept their arguments if applied to community museums with low overheads and a mission focused on local community needs (such as one finds in many emerging countries in the Asia, Africa and Latin America).  These exist at small scale around the world and likely can (and should) be governed from a commons standpoint of local engagement, control and financing in which the critical rules are devised by locals for the benefit of locals. These are institutions, however, with limited audiences and financial needs.  I also could extend their model, with adjustments, to institutions that may be owned and funded by governments, but only by governments that are tolerant of innovative uses of collections and spaces to serve local community needs.  Nina Simon’s vision for the “Participatory Museum,” based on her experience at the city-owned by autonomous museum in Santa Cruz, California, illustrates many of these principles. Hers, however, was a free-entry museum in a very “liberal” community.  Many museums in the US and abroad are experimenting with community-curated exhibits and projects, but that is not the same thing as being community-driven.  Indeed, the larger and more prominent the GLAM enterprise, the more that governance realities intrude on an idealized version of how a “commons-oriented” GLAM would work.

**Response:** We are truly grateful for reading our work and for raising all these insightful points so constructively. First of all, we totally agree that our conceptual framework cannot possibly fit with or otherwise accommodate all types of GLAMs. In fact, when drafting this article, we had in mind that our analysis applies to smaller institutions, which are best positioned to pursue and foster a commoning ethos. This is something that, admittedly, we had omitted to clarify eloquently in the first version of the paper - so thank you very much for bringing this up. Related amendments can be now found at the end of page 7, (‘These practices resonate with a commoning culture and can ‘push’ small and medium-size institutions struggling with limited finances and audiences towards a more organised ‘commons’ direction’) and in more detail, on page 12, “Towards exploring the application, flexibility and adaptability of the framework presented in this paper…” Regarding the distinction between commoning and tokenistic participation, we agree that this is indeed critical. We now raise this issue at the end of page 3, with a reference also to the work of Nina Simon (2010): “Although GLAMs are increasingly experimenting with community-curated exhibits and projects, they are still not necessary community-driven, especially when audiences are invited to take part in an institutionally-controlled process (Simon, 2010).These approaches have been criticised for using commons theory to describe projects that are participatory but not actually ‘commons’ (e.g. Lekakis, 2020; Iaione et al., 2022). As Dalfovo (2020) stressed, coining participatory approaches as ‘cultural commons’ risks the danger of unwillingly encouraging ‘commons-washing’ practices by ‘feeding the misleading paradigm of creative and cultural processes being the output of voluntary actions carried out for one’s own pleasure and in one’s own ‘free time’ without the respective compensation of mutual benefits”. At the end of our literature review (page 4), we have adapted the text to reflect your concern about overemphasing commons potential while underweighting governance considerations: ‘Overall, despite the growing interest in conceptualising culture and heritage as commons, there is still relatively limited work in the organisation and management of related resources as commons by memory institutions, such as GLAMs (Gould 2017, Lekakis 2020, Lekakis & Dragouni 2020). It is therefore necessary to delve deeper into the governance/management patterns and the design principles of cultural/heritage commons in order to address the ontology of these systems (i.e. resources, communities and rules of organising everyday work and strategy). This would be vital for informing our enquiry of how GLAMs could function as commons-oriented organisations. Yet, to avoid overestimating GLAMs’ potential to be governed as commons while underestimating managerial and political realities of their operation - from funding and long-term engagement to control and power issues over the uses of the past - it would be important to consider some of their distinctive features (e.g. organisation size, broader networks and relationships).’ Similarly, we rephrased the sentence ‘it is clear that GLAMs fall into the category of new commons’ (page 4), to point towards the conceptual - prefigurative character of our framework. Your feedback also led us to reflect more on the political and sectoral realities of managing heritage more collaboratively and realising a paradigm shift from centralised top-down heritage management to a more participatory model. As we argue on page 11: ‘a transition from the current canon of centralised heritage management to a commons governance would require devolving management authority from the top to the bottom, thus challenging long-held political, legal, economic and attitudinal realities across the sector. At organisational level, well-established GLAMs would be most likely reluctant to share their materials and knowledge in a commons setting, where they will have limited control and authority (Marttila & Botero, 2017). Thus, a commoning ethos necessitates a negotiation of the professionals and experts’ role as the  primary custodians of culture/heritage resources (e.g. collections, monuments, archives) so that commoners can be granted legitimate authority to act upon the management and governing of GLAMs.  Given that ‘community actors need to be proactively involved in design and co-management’ (Iaione et al., 2022), they need to work closely with GLAM professionals with the view to build a common ‘language’, channels for reciprocal knowledge transfer and mutual understanding so that the roles and areas of contribution can be co-decided and re-coded for each party. From a pragmatic point of view, GLAMs that are missioned to protect national ‘treasures’ and present dominant versions of the past have little flexibility to accommodate commons practices as compared to institutions specializing for instance, in local histories, sub-cultures, rural heritage or contemporary archaeology.’  

**Comment 2: ** In the case of large institutions (think the major Universal Museums, civically owned museums, libraries and so forth), the stakeholders engaged in these institutions are numerous (government owners, often separate government funders, philanthropic donors, professionalized staff members, sophisticated administrators, lenders and other financial overseers).  That complexity essentially makes the idealized notion of community-led, commons-oriented governance impossible.  Participatory measures that are taken may be token, and obstacles to community control impossible to surmount, especially where budgets are measured in millions of dollars or the equivalent. Such institutions, realistically, are unlikely to be “re-assembled in ways that will enable increased possibilities for museum staff and communities to assert control” (p7). Indeed, in many cases the professional staffs themselves would be opposed to delegating curation and presentation to non-“expert” community members. The authors suggest solutions, such as the representation of community members on governing boards or management and that rules and norms be assembled in a self-regulated manner. While the former is possible, it hardly emulates the sorts of self-organization and self-regulation envisioned in the commons literature. The latter, I would argue is exceedingly unlikely outside of small, local contexts. Legal ownership, fiduciary responsibility of directors, labour relations, and access all militate against a radical commoning of larger-scale and complex GLAM governance. It is not enough to say these things must change, as the authors do. They need to confront the reality that these changes fly in the face of substantial political, legal, economic and attitudinal realities. Not the least of these is that larger scale GLAMs require enormous amounts of money to operate, entitling the providers of such money (governments or philanthropists) almost insurmountable leverage over the conduct of the GLAM institution.

**Response: **We included two paragraphs right after the presentation of the framework and before the conclusions, discussing in detail issues of application, flexibility and adaptability of the framework presented in the paper.  

**Comment 3:** I also am concerned that the authors have not acknowledged the contestation within communities themselves. Often “communities” based on geographic proximity are highly discordant in their values, aims and interests. One consequence of an idealized view of communities is that it tends to produce an expectation that “community generated” content will be more inclusive of differences and expressive of tolerance. There are, in fact, no guarantees of this outcome. Communities around the world are notoriously not homogeneous, but it is commonplace in scholarly literature to act as if they are.  Ostrom’s work i demonstrates the need for communities to resolve such differences and admits of the difficulty of doing so as commons institutions increase in scale.

**Response: **Thank you very much for the insightful comments on the contested nature of (each and every) community. We address in the revised manuscript, the notion of community in two ways. First, the ‘whose heritage/whose community’ questions were also among our concerns while working on this framework. Considering the limited empirical evidence at hand, it is perhaps more safe to say that community formation processes are context-specific and idiosyncratic, so it would be hard to present a solid explanation about their operation. What we observe, and now mention in the revised manuscript, is that commons-oriented projects in the sector could emerge as a reaction to a current situation/problem (as in the case of Teatro Valley) or they may take a more proactive approach, e.g. as in citizen groups doing oral history. Please see our amendments on page 4: ‘In this light, heritage commons have been envisioned as dynamic and porous systems that protect the resources at hand while catering for the common benefit of involved communities (Lekakis, 2020). In such systems, cultural heritage can be understood as a resource that is compiled collectively, as part of the work performed by memory institutions operating in the GLAM sector, to promote greater representation and more democratic/multivocal discourses about the past. In line with other commons, this ‘collective production’ of cultural/heritage commons can bring together a community of commoners (Bailey and Marcucci, 2014). But, as scholarly work has emphasised, ‘communities’ (geographic, virtual or imaginative) are not always ‘community-like’ but rather incohesive assemblages of people with divergent interests (Waterton and Smith, 2010). Thus, communities with interest in the cultural/heritage resource shall be regarded as plural and geographically-unbound, and they may include culture professionals, art workers, heritage researchers, local residents, patrons and many others (Bailey and Marcucci, 2014). These communities are likely to be formed dynamically, reacting against the neglect or even hostility of public authorities (e.g. Teatro Valley in Italy; Bailey and Marcucci, 2014), or they may take a proactive approach to heritage-making (e.g. grassroots oral history projects, such as the ‘Oral History Groups’ in Greece’). Additionally, we have included a more explicit theorisation of the contested nature of community, making reference to the notions of “agonistic pluralism” and “agonistic commons” at the end of the literature review, on pg. 4 ‘More fundamentally, and beyond the relations of communities to other, ‘external’, actors, as Bruncevic (2018, p. 143) argues in her analysis of the fluid and contested nature of community and property of cultural artefacts “one of the more significant problems with the concept of community is that it is often presupposed as homogeneous or at least as an, at any given point, definable group”. As, however, heterogeneity of actors and potential divergence of interests are characteristic of contemporary complex communities, conflict within the community over what may be considered cultural resources and how these may be optimally managed becomes a central issue to be addressed through collective commoning practices. Recognising that divergence and adversarious positions are inherent in communities, we may conceive of “agonistic pluralism” (Mouffe, 1999, 2007) as an essential element of community building, and recognise cultural commons as “agonistic commons” (Kioupkiolis, 2019), which could “nurture bonds of mutual recognition and coalescence among different parties who remain rivals along certain dimensions, making more space for plural freedom under conditions of dissent and division” (Kioupkiolis, 2019, p. 28). It is not, therefore, the existence of divergence and adversariality per se which is antithetical to the notion and practice of community, or to the practices of commoning devised by a community, but the management of such divergence in ways which will not presuppose the homogeneity or even uniformity of participating actors and entities. This brings to the fore the importance of institutions and processes of governance.’  

**Comment 4:** The authors identify three “key principles that determine GLAMs’ potential to operate as commons: (a) governance, (b) autonomy and (c) accessibility” (p10). My essential recommendation for this paper is that the authors ponder the implications of the arguments I am presenting and either offer a more nuanced evaluation of how the realities of scale, ownership/management structures, and financial dependence require them to modify their arguments and/or suggest how, in the context of real-world GLAM operations, they could feasibly adapt larger-scale GLAMs to overcome the limitations in governance, autonomy and accessibility that interfere with their vision of a commons-oriented GLAM sector.

**Response: **Taking into consideration the realities of scale, management structures and financial challenges/dependencies, we now offer a new conceptualisation of how the key principles can be seen in the case of GLAMs. What we say is that there are very few GLAMs organisations that operate as commons per se, meaning that they fulfil all three criteria and principles. Most of the GLAMs use commoning practices, but cannot be considered as commons per se. Looking at the commoning practices of GLAMs we see that many large and established GLAMs entities (either publicly owned or private institutions), as well as smaller organisations, use a lot of co-creation practices and are much accessible through offering large parts of their collections for free to the public through digital means. Thus, most often GLAMs “enter” the schema from above and usually stay at the first level of accessibility. On the other hand, there are fewer GLAMs that go into the second level, as they are challenged by economic stagnation and they try to find solutions towards financial sustainability through commoning practices (crowdfunding, etc). Usually these are small organisations that are not financially backed by public spending, or there are organisations that rely a lot on volunteering labour. Moreover, fewer ones can be found in the third level, where we usually find horizontal decision-making governance systems (through assemblies etc), collective ownership etc. Thus, we can generally say that the three principles of commons-oriented GLAMs can be used as a conceptual framework to evaluate the degree of commoning practices in GLAMs, where the real-world experience shows that most of GLAMs enter that schema from above, and usually do not go on to the second and third level (usually public and large GLAMs) and there are very few other GLAMs (usually more community-led) that enter the schema from the bottom and use commoning practices from the three levels in order to overcome specific challenges and operate as commons-oriented.
